# Infection from the Mouth

**Published:** 1894-05

**Authors:** E. L. Clifford

**Affiliations:** Chicago


### THE DENTAL REGISTER.

VOL. XLVIII.] MAY, 1894. [No. 5.

### COMMUNICATIONS.

### Infection from the Mouth.

Read in the Section on Dental and Oral Surgery, at the Forty-fourth Annual Meeting of the American Medical Association.

BY E. L. CLIFFORD, D.D.S., CHICAGO.

[*Continued from April Number, page* 198.]

Now, as to the clinical, pathological and practical aspects—according to the different sources of poisoning, or intoxication, as it is technically called, there are correspondingly different indications, signs or symptoms which Dr. Brown classifies as follows: 1, poisoning by the extractives is attended by *hyper*-thermia; 2, poisoning by "animal alkaloids" is accompanied by *hypo*-thermia; 3, a combination or succession of *hyper*-thermic and *hypo*-thermic phenomena may become manifest, according to the combination or alternation of poisoning by the deleterious physiologic products, or their antagonistic action. Some contradictory evidences of the above are accounted for as follows: "Where 'extractive matters' accumulate in the blood we detect hyperthermia; on the other hand, if 'alkaloids' accumulate, we have hypothermia, while if the two factors co-exist they may neutralize each other, or become antagonistic in their action, so that temperature may remain stationary or normal. But should one or the other predominate, immediately the scale is turned, so that some variation may be noted. In this auto-infection, this spontaneous or self-infection, of the living organism by the alkaloids and extractives of its own formation there is no question of quality, but simply one of quantity to be considered, by reason of the essential physiologic source and action of the poison. In other words, the healthy living organism may become poisoned (more or less slowly) by the accumulation within itself of deleterious substances normally elaborated, but imperfectly or defectively eliminated. Hence the slow and insidious onset of much ill-health, and from which recovery is correspondingly slow." Now, in what way does this auto-infection of the system take place? Brown tells us that it can only be understood and explained by the mode in which we regard the phenomena of life. Life is a ceaseless decay with a ceaseless repair. "Normal health is conditioned on an incessant formation, transformation and elimination of the effete or old organic materials which must give place to new. It is this effete material, which, therefore, represents a series of partial deaths, and which, as the result of organic functional operations, constitutes life, during which the tissues and organs in the processes of their metabolic changes perform a constant function of disintegration, fabricating during these processes those alkaloids and extractives, those x, y, z's of pathology which must be regarded as veritable '*physiologic ashes*' (Brunton), resulting from the processes of combustion of the elements of organic tissues."

Having progressed thus far, and established the fact, we think, of the presence and power of these poisonous agents, let us for a while cast a glance at the oral cavity of man, and see if any conclusions can be drawn as to the part it plays in promoting or preventing the formation of these factors in the causation of disease.

1. We will claim that no portion of the organism is of more importance, or is more active as a veritable chemic laboratory, than the mouth. It is established, that in the processes of putrefactive decomposition, certain alkaloids are elaborated from the proteid substances. We all know that in the cleanest mouths certain portions of these proteid substances will remain, as they are ingested with the food we take, as well as the water we drink, and the air we breathe. We know also that in the cleanest and healthiest mouths, absence of bacteriologic influences is but an utter impossibility, and that these micro-organic agencies, furnished as they are with the proper media or food, conjoined with a suitable temperature and abundant moisture, make the mouth one of the best incubators that can be imagined. If this be so in mouths that have been no strangers to proper hygienic influences, how much more strongly is the fact established of the influence of this territory, when carelessness and neglect have been a predominant feature? And, if we accept the theory of Aitken that these alkaloidal products vary as to their poisoning power according to the stage of decay at which they are produced, how important is it that these hygienic influences be enjoined and enforced. Then again, accepting the dictum of Gautier, that these animal alkaloids can not be excluded from the general economy, and that they are even necessary as a product of vital physiologic processes, it certainly becomes the duty of that branch of medical science to which the very entrance of the digestive tract is allotted for control and treatment, to see to it that contaminating influences are eliminated. Again, if the possibility of the formation of these animal alkaloids is acknowledged without the aid of bacteriologic fermentation, as shown by Gautier, how much more deleterious must be their effects, and how much greater must be their production when conjoined to and assisted by these physiologic agencies. If an abnormal amount of these poisonous substances is produced within the intestines when in any way disordered, how much more reasonable to conceive that the manufacturing capacity of the mouth is greatly increased, when a condition that might be termed absolute filth is no stranger to the eye of the observant dental practitioner. If the amount of these agencies formed in the intestines of a healthy man in twenty-four hours is sufficient to kill him if absorbed and secretion stopped, it will require no great stretch of your imagination to realize that enough poison could be taken from the mouth of the average dental patient to kill a small army of inoculated subjects if properly applied. Let the physician who doubts this statement recall the condition of wounds, and ponder over the difficulties of his prognosis when called to attend a traumatism caused by the human bite. The conclusions of

Dr. Brunton will also go far to account for the thermal changes which take place and accompany these traumatisms, when viewed from a clinical or pathological standpoint. Miller establishes well the part played by the human mouth as a generator of micro-organic life, and his testimony is corroborated by other investigators; not only are large quantities of the animal alkaloids produced within the intestines and other portions of the alimentary tract, but large quantities are swallowed with innumerable bacteria which have already been produced in the mouth, and, as stated by Miller, they give rise to local and general disorders of the most serious nature, produced partly by the direct action of the micro-organisms, and their products upon the teeth and the mucous membrane of the mouth, partly by swallowing large portions of bacteria, partly by carrying them into the lungs, particularly in cases of violent inspiration, and finally by their obtaining an entrance into the blood or lymph vessels in various ways, such as by a breach in the continuity of the mucous membrane brought about by mechanical injuries (wounds, extractions, etc.), through the medium of gangrenous tooth pulps, which usually lead to abscess at the point of infection; sometimes with secondary septicemia and pyemia with fatal termination, by resorption of poisonous waste products formed by bacteria; by the inspiration of particles of slime, small pieces of tartar, etc., containing bacteria, and by contact with the oral and pharyngeal cavities, whose power of resistance has been impaired by debilitating diseases, mechanical injuries, etc.

The very antechamber of man's vegetative existence, the very portal of the human body, furnishing as it does a most excellent nursery for these etiologic factors, has not received from the general profession of medicine, within the past, that attention which its importance has deserved; but, thanks to the work and investigations of some of the members of our specialty, the physician can no longer ignore these factors and feel that he has done justice to himself and the patient who has so confidently placed himself under his care. The results of the last few years have proved that if many of the diseases whose origin is enveloped in mystery could be traced to their source, they could be found to have originated within the oral cavity. And, as to the danger of such diseases as do follow from infection traced directly to the mouth, it seems there could be but one thought, when we reflect that out of a carefully prepared table of 149 cases by Miller, there resulted 50 deaths, 19 cases of syphilis, 2 cases of blindness and 2 cases of a loss of a part or the whole of one of the maxilla.

Most of the work which has been done up to this time (with the exception of that by Miller) has been with a view of attracting the attention of the dental specialist to infection which has been carried *into* the mouth from without, resulting in local manifestations which would naturally come under the supervision and treatment of the dentist. Miller has opened up, and is cultivating with skill and determination a new field of research, and it seems to be his object, as it is mine at this time, to attract the attention of the general practitioner to infections which are carried *from* the mouth and are planted in such soils as will prove favorable to their future germination and growth, hence, proving an obstacle in the path of him who would assume to treat these pathologic lesions, without due appreciation of the important part these agencies and this location play as factors in etiology. It would not be difficult to convince the observant dentist of the truths here implied; it would only be necessary, were that our object, to ask him to recall from his every-day experience ample facts to substantiate the position. He has seen many times the general health of his patients vastly improved by a proper care and attention of the mouth. He has studied, as a specialist, the chemic powers of decayed teeth, vitiated and poisoned secretions, and fully appreciates their power and influence, but I am forced to the belief that the physician of the past has either willfully or ignorantly ignored the important part this portal plays in the aggravation of diseases, or as a preventive of cure. And this thought is only enhanced and enlarged by the recent practices of the more observant and faithful, in calling to their assistance the aid of the competent orist, in assisting him to re-establish physiologic perfection.

To make the attempt to enumerate and to classify the different ailments which are either caused or aggravated by the unhealthy and unwholesome condition of the mouth, would be too great a task for one paper, if it were necessary, but it is not. Miller has done and is doing this work for us, and it is our province to assist as much as possible in bringing to the notice of the profession the excellence of his work and the importance of his conclusions. At such times as this, we can but generalize. Every physician of experience has often, no doubt, found himself in the dilemma of not being able to account for the cause of many cases in practice—and while I would not have him look to the mouth as the harbinger of all these causes, I would ask his attention to that sphere, as a possible, and I might say, a probable source in many cases.

There is no doubt that the nervous system in many instances, and by very remote manifestations, is often injured to an alarming extent by the mechanical or chemical irritation emanating from an oral organ. The alimentary tract, we know, is often deprived of the privilege of doing its proper duty by contaminations implanted into the first bolus of food, started on its course through a filthy and poisonous mouth, and added to the chemic contamination may well be mentioned the mechanical, which in cases of malocclusion, tender and sore teeth, or edentulous mouths prevent a proper mastication of food, entailing upon the stomach an office and a duty never designed by nature. This overwork of the stomach, combined as it often is with the chemic poisons of the diseased mouth, of course is sufficient to interrupt the proper function of other organs in the tract as they are reached, and consequently the whole canal becomes a source of irritation and of poison. Now, the primary source of health and growth being so far drawn from the path of rectitude laid out to furnish man with ease and not disease, it is evident that future digestion, assimilation and nutrition is only a matter of impossibility. If the ptomaines, leucomaines and extractives are found in every organism, and under all conditions, it is only rational to believe that an all-wise Providence has designed them for a specific and for a physiologic purpose. They have a function to perform, even though it were only that of scavengers for other tissues. It would certainly not be wise to place a minor importance upon the function of the kidneys, simply because their principal office seems to be that of eliminators—on the contrary, the more reason why their integrity should be preserved and their powers not deteriorated, in order that those substances which seek this channel may be thrown off and not left to accumulate, to contaminate and to poison what would remain healthy.

Likewise, if micro-organisms are so universally present, the same facts would equally apply to them, and it is to the prevention of a growth of an abnormal quantity, and to the prevention of the introduction into the organism of specific germs, that the attention of the therapist of the future will be more particularly directed. If we could only get our medical confreres to pay more attention to the chairs of oral and dental pathology that have lately been introduced into our colleges, to the sections on these subjects that have been attached to all the congresses and national associations of late years, we believe they would more fully appreciate their importance, would more often seek the advice and assistance of the specialist, and this in turn would create a bond of union between us and them, stronger and more appreciated than any that has existed up to this time.

Aside from local inoculations and their results, which are usually classed among dental diseases, there are many forms of aberrations that the condition of the mouth may be held especially responsible for. Paralyses, pareses, anesthesias and hyperesthesias adorn the border line where it is difficult to determine whether the case is one for the specialist or the general practitioner; but these lesions, having long been noticed as doubtful in their etiology, are not classed among the obscure, and are often brought to the notice of the specialist and his assistance sought. But in those more obscure cases where the cause is greatly clouded, mistakes are more liable to occur, and the physician often battles vainly in his efforts to restore a healthy status by the ever interfering and constantly infecting products of this portal to man's existence. Miller has shown that pulmonary and bronchial diseases are excited or aggravated by the inspiration of germs from the oral cavity, as evidenced in the case cited of J. Israel, where primary actino-mycotic infection followed the lodgment of a small piece of tartar within the lung. Several cases within my own practice confirmed the theory, and I have more than once seen the benefit, and been able to bring it forcibly to the notice of the attending physician, where marked improvement followed the sterilization of the mouth, when all efforts had proved fruitless prior to this step. Alimentary disorders are of such common occurrence, as a result of defective and contaminating mastication, as to require no extended notice, and from this one condition all other organs are more or less affected, both nervous and vascular. Bednar, said to be the first to give clear expression to this view in 1854, states that, "indigestion may be brought about *directly*, by taking into the stomach any substance already in a state of fermentation; *indirectly*, when the food taken into the stomach undergoes subsequent fermentation on account of its disproportion of the gastric juices." Henoch favored this conception and found in it a cause for a large number of diarrhœas, while Minkowski classsifies under five heads the disturbances directly caused by the fermentative processes in the stomach:

1. Substances may be formed which irritate the mucous membrane of the stomach, and bring about a state of catarrhal inflammation.

2. Considerable quantities of gas may be found heightening the mechanical insufficiency of the stomach.

3. Fermentations may lead to the production of substances having toxic properties.

4. In the fermentation of albuminous substances, alkalin products may arise which neutralize the gastric juice.

5. The gastric fermentations may exert a great influence on the functions of the intestines.

And now, what is our lesson? We would ask the general practitioner to regard the unhealthy condition of the oral cavity, with all its contents, as an important factor in the causation or aggravation of diseases, having shown, as we believe, that within its boundaries lie the primary causes of many pathologic lesions; that good, healthy, clean and serviceable dental organs are absolutely essential to the enjoyment of physiologic ease, and that in many cases the dental specialist has within his power the rendering of valuable assistance in the search for this result.

DISCUSSION.

Dr. J. S. Marshall said that the paper opened up a field of thought to all dentists, and the application made to the diseases of the general system will make it valuable to the general practitioner. Almost every pathogenic micro-organism has been found in the mouth by Dr. Miller, and we can not be too careful about introducing them into our own systems when operating. Upon two occasions he had pricked his finger while at work, and each time had troublesome sores from the accidents, showing that there was more than a mere wound to deal with.

Dr. Talbot said there were, no doubt, many cases where a foul condition of the mouth was responsible for troubles in the general system, and where an improvement in health and increase in weight had followed the putting in order of the mouth. The infection from the mouth of a patient, too, had often caused trouble to the dentist, such as inflammation of the eyes and perhaps sore throat, consumption, and tonsillitis.

Dr. Taft said we should promote the function of the eliminative organs; the skin should be kept clean, the liver active, the kidneys, lungs, etc., in good condition, and then there will be less likelihood of infection from bacteria. Care should be taken to regulate the food supply as to quantity, quality, and the proper preparation; these precautions, with reasonable effort toward keeping the mouth clean, will be sufficient to guard against these infections. He also spoke of the habit of mouth-breathing, and said dentists should impose on all their patients the necessity of keeping the mouth closed as much as possible.

Dr. Clifford said that the main idea in his paper was to call attention to the effect the micro-organisms of the mouth had upon the general condition. Dr. Taft spoke of keeping the eliminating organs in good condition, but this can not be done unless the mouth is kept clean, for if the mouth contains enough poison to keep the blood, liver, and skin in bad order, they can not be got right until the mouth is rid of the cause of the trouble.

